# How safe is too safe? A dual-pathway framework for understanding the dark side of psychological safety

**DOI:** 10.3389/fpsyg.2026.1879175

**Published:** 2026-08-18

**Authors:** Francesco Cangiano

**Affiliations:** 1 Department of Defense and Security, Rabdan Academy, Abu Dhabi, United Arab Emirates; 2 Bond Business School, Bond University, Robina, QLD, Australia

**Keywords:** dark side, psychological safety, regulatory loss, self-regulation, team performance, voice behavior

## Abstract

Psychological safety has become one of the most celebrated constructs in organizational behavior, consistently linked to voice, learning, and team effectiveness. This paper challenges the assumption that reducing interpersonal risk is universally beneficial. Psychological safety operates through two competing pathways: one that enables expression and participation, and one that weakens the interpersonal evaluative pressure, self-monitoring, and norm adherence that sustain coordination and performance. This second mechanism is termed the regulatory loss pathway. The pathway operates through three behavioral channels: the loss of functional filtering, effort withdrawal, and norm erosion. Drawing on self-regulation theory, accountability research, and the social loafing literature, the paper develops a dual-pathway model that specifies when psychological safety helps and when it hurts. Evidence from military operations, aviation, and high-reliability organizations shows that some of the constraints psychological safety removes serve as structural conditions for coordinated and timely decisions. The model predicts that the relationship between psychological safety and performance is nonlinear, with moderate levels producing the best outcomes and very high levels generating process losses, particularly where coordination demands are high and external accountability is weak. The framework also specifies how hierarchy and power asymmetries shape both pathways. Seven testable propositions and key boundary conditions are developed, with implications for high-stakes organizational design.

## Introduction

On January 28, 1986, the Space Shuttle Challenger broke apart 73 s after launch, killing all seven crew members. Engineers at Morton Thiokol had raised concerns about O-ring performance in cold temperatures the night before the launch; the warnings were heard, debated, and overridden. What silenced further pressure was less the engineering evidence than the interpersonal cost of pressing the point upward. Cases like Challenger have made a powerful case for psychological safety as a condition for effective organizational decision-making.

Since Edmondson’s (1999) foundational work, psychological safety, defined as the shared belief that a team is safe for interpersonal risk-taking, has accumulated one of the most robust evidence bases in organizational behavior. It has been consistently associated with voice behavior, team learning, creativity, error reporting, and performance across healthcare, education, and corporate contexts (Frazier et al., 2017; Newman et al., 2017). Subsequently, the construct moved rapidly from academic journals into leadership development programs and organizational culture assessments. The overarching message became that reducing interpersonal risk improves performance. This consensus, however, is based on a narrower empirical foundation than is commonly acknowledged. Most of the evidence has been generated in environments characterized by moderate stakes, relatively flat hierarchies, and a cultural emphasis on participation and inclusion, settings where the benefits of expressive communication make intuitive sense (Edmondson and Lei, 2014; Baer and Frese, 2003). The problem is that a significant class of organizations looks nothing like this. For instance, military units, emergency response teams, nuclear operations, and surgical theaters operate under conditions that are categorically different: extreme time pressure, formal and legally enforced hierarchy, high interdependence, and communication requirements that prioritize coordination over open-ended expression. In these settings, speaking up is only part of the issue. The content, timing, and volume of what is said determine whether voice supports or disrupts the functioning of the system as a whole (Weick and Sutcliffe, 2015; Snook, 2000).

The distinction matters more than the literature has recognized. Reviewing 25 years of research on psychological safety, Edmondson and Bransby (2023) note that the field has made substantial progress in documenting enabling effects but has given comparatively little attention to boundary conditions and potential costs. Eldor et al. (2023) in one of the first empirical examinations of this question, found that the relationship between psychological safety climate and in-role performance is nonlinear: moderate levels predict better outcomes, but very high levels are associated with performance decline, particularly in routine task environments. Their finding points toward something the dominant literature has arguably been slow to address: the removal of interpersonal constraint is not uniformly beneficial, because some of those constraints serve regulatory rather than merely inhibitory functions.

In military operational planning, encouraging voice and the challenging of assumptions during the deliberation phase is genuinely valuable because it surfaces new information, stress-tests plans, produces alternatives, and reduces premature interpretive closure (Tetlock et al., 2014). However, the same dynamic during execution can introduce coordination failures and undermine the command authority on which rapid, collective action depends (Boin et al., 2017). Commercial aviation confronted the costs of silence after the 1977 Tenerife disaster, in which 583 people died partly because the prevailing communication culture left junior crew members unable to effectively challenge a senior captain’s authority. The industry’s response, crew resource management, engineered a calibrated version of psychological safety: crew members were trained to challenge decisions at defined moments, while command authority and operational discipline stayed intact (Helmreich and Foushee, 1993). Thus, the same regulatory regime that trained junior officers to challenge captains also prohibits nonessential communication during critical phases of flight, restricting voice precisely where its costs to coordination are highest (Flight Crewmember Duties, 2024). Psychological safety research has theorized the first half of this design and not the second: some constraints on communication are functional, and removing them has costs.

The present paper offers a conceptual framework to address this gap. Psychological safety operates through two simultaneous pathways. The first is well established: by reducing interpersonal risk, it enables voice, engagement, and learning. The second has received far less theoretical attention. Interpersonal risk does more than inhibit people; the anticipation of being judged is also what makes them prepare before speaking, weigh whether a contribution is worth the group’s time, and hold themselves to shared standards. Reducing that risk enables voice; removing it may also weaken the self-monitoring and accountability that keep contributions disciplined, decisions timely, and coordination intact. This paper terms this second mechanism the regulatory loss pathway. Its significance has been obscured by a literature that has treated constraint largely as an obstacle to performance rather than as one of its structural conditions.

The analysis proceeds in four steps: a review of the established evidence on psychological safety and its enabling effects; a reframing of the construct as a mechanism of interpersonal risk reduction with inherently dual consequences; the development of the regulatory loss pathway and its behavioral channels; and the integration of both pathways into a model of divergent outcomes, with boundary conditions that determine which pathway dominates and a set of testable propositions. The paper closes with theoretical contributions, practical implications for high-stakes organizational contexts, and directions for future research.

The central contribution is a more complete account of psychological safety, covering both its enabling effects and the regulatory costs that accompany them. Understanding the enabling effects allows organizations to reduce the cost of silence. Understanding the regulatory costs allows them to design environments in which speaking up supports rather than disrupts performance.

## The established view and its limits

The case for psychological safety rests on a robust empirical foundation. Since Kahn's (1990) foundational theorizing and Edmondson's (1999) operationalization at the team level, research has consistently shown that reducing interpersonal risk encourages voice, learning, error reporting, and participation across industries, national contexts, and levels of analysis (Frazier et al., 2017; Newman et al., 2017; Nembhard and Edmondson, 2006; Baer and Frese, 2003). The enabling mechanism is well understood: when individuals do not fear embarrassment or reputational damage, they contribute more, and those contributions improve collective outcomes.

This success has come at a theoretical cost. The strength and consistency of the evidence base has encouraged a tendency to treat psychological safety as an unambiguously positive construct that organizations should cultivate as extensively as possible. The dominant research agenda has focused accordingly on identifying antecedents and mechanisms rather than downsides or boundary conditions (Edmondson and Bransby, 2023). The regulatory functions that interpersonal risk may serve, prompting individuals to filter contributions, calibrate timing, and maintain communicative discipline, have gone largely untheorized.

This imbalance is partly contextual. Much of the evidence comes from settings with moderate stakes, collaborative tasks, and flat hierarchies, where constraints on voice are plausibly dysfunctional and the benefits of participation are easy to observe. It does not travel well to military units, surgical teams, or nuclear operations, where communication must be open, but also disciplined, timely, and filtered. Supporting this, a meta-analysis by Sanner and Bunderson (2015) showed that psychological safety’s benefits are significantly stronger in knowledge-intensive settings than in routine or execution-oriented ones. Eldor et al. (2023) found the relationship with performance to be nonlinear, with moderate levels producing the best outcomes and very high levels predicting decline, particularly on routine tasks. What is missing is a theoretical account of the conditions that determine which effect dominates.

## Reframing the construct: interpersonal risk reduction and its dual consequences

What does psychological safety actually do to the conditions that regulate expression, beyond lowering the barriers that suppress it? At its core, psychological safety reduces the perceived interpersonal costs associated with speaking up and challenging others. Rather than treating this reduction as inherently positive, in this paper psychological safety is presented more neutrally as a mechanism of interpersonal risk reduction. This redirects theoretical attention from outcomes to process, and from the question of whether people speak to the question of how the anticipation of social consequences shapes what they say and how they say it.

From this perspective, psychological safety produces two simultaneous consequences that may operate in competing directions. The first is the enabling pathway, documented extensively in prior research: psychological safety lowers the social costs of participation, thus bringing into deliberation ideas and information that would otherwise remain unvoiced (Edmondson, 1999; Kahn, 1990; Morrison, 2014). The second is less familiar. The two pathways are not independent processes, and they are not opposite ends of a single continuum. They are two consequences of one mechanism. The anticipation of interpersonal evaluation performs two functions at once: it inhibits expression, and it disciplines it. Reducing that anticipation does both at the same time. When individuals anticipate social evaluation and its potential consequences, they are both inhibited from speaking and prompted to regulate what they say. They filter contributions for relevance, calibrate timing, and align their behavior with group norms (Goffman, 1959; Schlenker and Leary, 1982). These processes run on the anticipation of being evaluated, so they weaken as interpersonal risk approaches zero, along with the fear. The result is more voice, and potentially less disciplined voice. In contexts where coordination depends on the quality and timeliness of communication, this distinction matters considerably. The distinction anticipates a point developed later: more contributions are not automatically better contributions, given that removing evaluation apprehension raises the volume of contributions without raising their average quality (Diehl and Stroebe, 1987; Paulus and Yang, 2000), a pattern the voice quantity and voice quality subsection below treats as central to the regulatory loss pathway. This parallels arguments in the proactivity literature that not all proactive behavior is beneficial: voice and initiative that are poorly timed or misdirected can disrupt rather than improve performance, and the concept of wise proactivity captures precisely this idea that the value of speaking up depends as much on how and when it occurs as on whether it occurs at all (Wang et al., 2025).

The question, then, is what normally keeps voice disciplined, and what happens when that mechanism is weakened. Self-regulation describes the processes through which individuals monitor and adjust their behavior in relation to goals, standards, and social expectations (Carver and Scheier, 1998; Baumeister and Heatherton, 1996). These processes are not purely internal. They are also activated by external cues, including the anticipation of evaluation and the prospect of consequences. Research on evaluation apprehension has shown that the presence of an evaluative audience systematically affects the selectivity and effort with which individuals approach tasks (Geen, 1991; Bond, 1982; Szymanski and Harkins, 1987). Interpersonal risk functions as precisely this kind of regulatory signal: it prompts individuals to attend more carefully to the quality and appropriateness of their contributions. Evaluative pressure has two sources that the argument distinguishes throughout. Interpersonal sources include the anticipated judgment of peers and superiors in everyday interaction: the prospect of embarrassment, ridicule, or social sanction. Structural sources include formal evaluation, recorded review, and professional consequence, which operate whether or not the interpersonal climate is forgiving. Psychological safety attenuates the interpersonal sources. Whether the structural sources remain intact is a question of organizational design, and it is the question the boundary conditions address. The regulatory losses described below occur at very high levels of psychological safety, not at moderate ones. Moderate reductions in evaluative pressure leave these processes largely intact; the processes weaken when evaluative pressure becomes minimal. This asymmetry underlies the nonlinear relationship formalized in Proposition 6.

**Figure 1:**
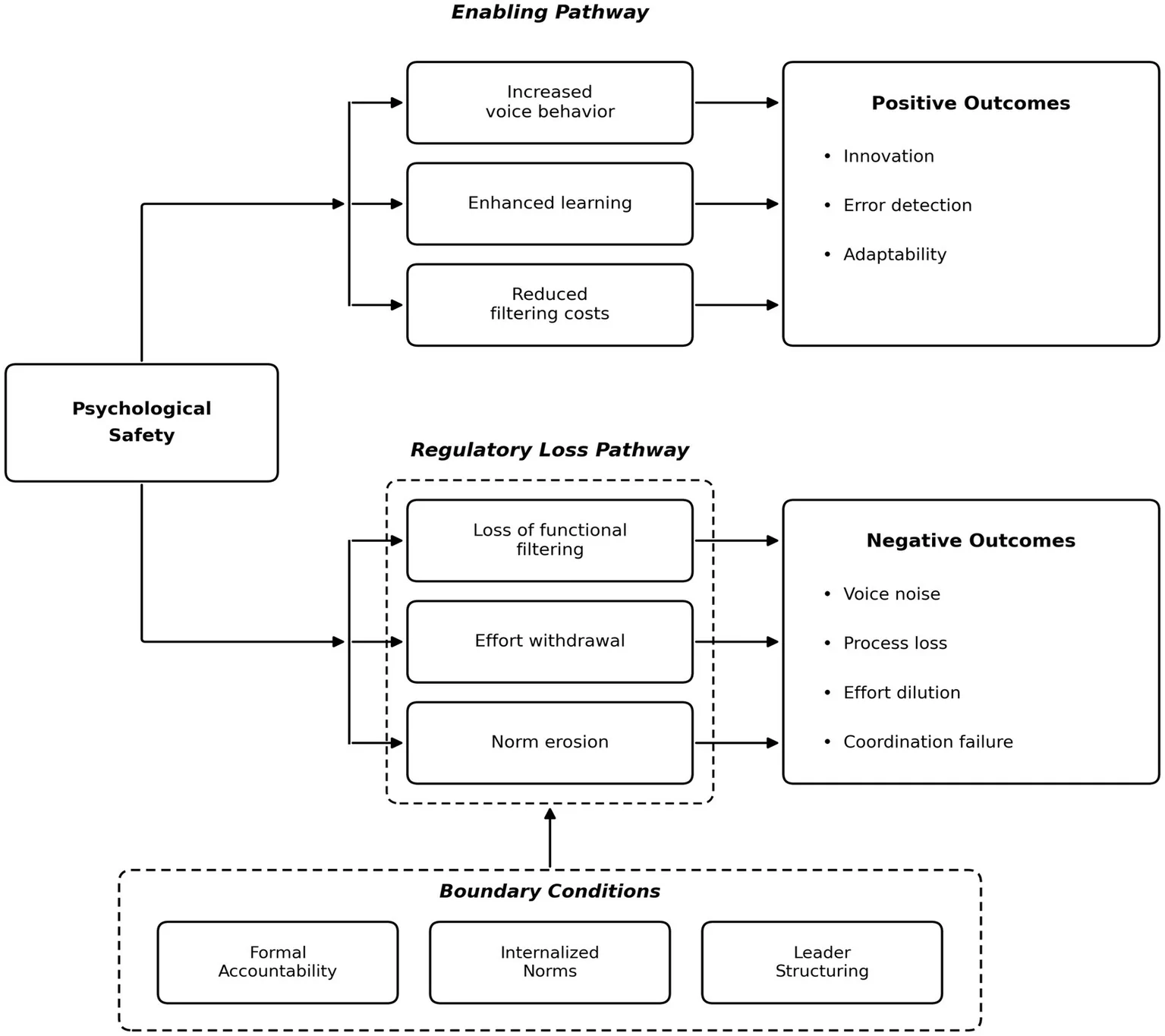
The dual-pathway model of psychological safety. Solid arrows show the causal sequence within each pathway. The dashed connector shows the boundary conditions acting on the regulatory loss pathway as a set.

Regulatory loss is not proposed as a construct that competes with accountability, self-monitoring, or social loafing. It names the pathway through which attenuated interpersonal evaluative pressure weakens three existing mechanisms at once: self-monitoring (Goffman, 1959; Schlenker and Leary, 1982), the anticipatory effort accountability research documents (Tetlock, 1992; Lerner and Tetlock, 1999), and the evaluation potential social loafing research treats as central to effort withdrawal (Karau and Williams, 1993). None of these three mechanisms is new. What accountability theory alone does not explain is why the same reduction in interpersonal risk that weakens them also increases willingness to contribute in the first place. Filtering loss, effort withdrawal, and norm erosion, the three channels in Figure 1, are how that weakening shows up behaviorally; process loss and coordination failure are its downstream, team-level outcomes. Regulatory loss names that full pathway. No single mechanism among the three explains the pairing on its own.

The literature on accountability makes the same point independently. Tetlock (1992) demonstrated that individuals who anticipate having to justify their decisions to others engage in more complex reasoning than those who do not. The anticipatory nature of this finding is important: it is not evaluation itself but the *expectation* of evaluation that drives more careful cognition and behavior. Lerner and Tetlock (1999) extended this work to show that accountability influences both decision quality and the effort and attention individuals invest in forming judgments, with pre-decisional accountability producing the strongest effects. Frink and Klimoski (1998), in a comprehensive review of accountability in organizational contexts, found that evaluative pressure shapes task performance, citizenship behavior, and ethical decision-making through these same anticipatory mechanisms. Applied to psychological safety, this implies that reducing evaluative pressure makes people more willing to contribute and may simultaneously reduce the cognitive and behavioral effort that anticipatory evaluation normally brings about.

The social loafing literature points to a similar conclusion from a different angle. Latané et al. (1979) demonstrated that individuals exert less effort on collective tasks when their individual contributions are not identifiable or evaluable, a finding replicated extensively across cultures and task types (Karau and Williams, 1993; Liden et al., 2004). The mechanisms underlying social loafing, reduced identifiability and diminished evaluation potential, overlap substantially with the conditions produced by high psychological safety. Psychological safety is not defined as tolerance of poor performance, and its proponents are explicit that it does not mean lowered standards (Edmondson, 2018). The bridge from loafing research to psychological safety therefore rests on a premise that should be stated: the perceived climate signal is coarser than the construct’s definition. Individuals cannot finely distinguish a climate in which honest errors carry no social penalty from one in which low-effort or poorly calibrated contributions carry none, so climates that mute the first signal tend to mute the second. Pearsall and Ellis (2011) provide direct evidence of this coarseness, showing that psychological safety lowered the perceived cost of norm violation broadly, beyond the cost of speaking up alone. When individuals believe that mistakes, low-effort contributions, or poorly calibrated input will not be penalized, the evaluative pressure that normally motivates careful preparation is attenuated.

Much of the psychological safety literature treats evaluative pressure and the norms that regulate communication as barriers to be minimized, and in contexts where voice is chronically suppressed, that framing is indeed appropriate. But in many organizational settings, constraints serve regulatory functions that have nothing to do with suppression. They structure when input is appropriate, filter what is worth saying, and maintain the coordination on which collective performance depends (Hackman, 2002; Weick and Sutcliffe, 2015; Feldman, 1984). The crew resource management protocols discussed in the introduction illustrate this directly: the industry calibrated psychological safety, preserving structured challenge at defined moments while maintaining the communicative discipline that coordinated decisions require (Helmreich and Foushee, 1993). That distinction, between willingness to speak and discipline in speaking, is the theoretical gap this paper addresses.

A useful anchor here is Edmondson’s own framework distinguishing psychological safety from accountability (Edmondson, 2018). In her model, high psychological safety combined with low accountability produces a comfort zone rather than a learning zone. The present argument extends this intuition by specifying the mechanism: low accountability weakens the self-regulatory processes through which evaluative pressure normally disciplines behavior. The implication is that psychological safety and accountability are structurally interdependent: without accountability, psychological safety may slowly undermine the conditions it is assumed to improve. It should be noted that this claim is conditional. Psychological safety does not lower formal standards or dismantle accountability structures, and nothing in this argument revises Edmondson’s (2018) point that it should not. The attenuation of interpersonal evaluative pressure changes how binding existing standards and structures feel. It does not change whether they exist. The negative outcomes developed in the sections that follow are accordingly most likely at very high levels of psychological safety, and specifically where the structural sources of evaluative pressure identified above, formal accountability, leader structuring, and internalized norms, are also weak. Where those structures remain strong, psychological safety and disciplined performance can coexist; the boundary conditions developed later in the paper specify when.

This reframing yields three foundational propositions, each capturing a distinct element of the dual-pathway mechanism. Proposition 1 reflects the well-established enabling effect: psychological safety is positively associated with willingness to engage in voice and interpersonal expression (Edmondson, 1999; Frazier et al., 2017). Proposition 2 captures the regulatory loss mechanism: psychological safety is negatively associated with interpersonally based evaluative pressure, resulting in reduced self-regulation of communication and behavior (Tetlock, 1992; Carver and Scheier, 1998; Baumeister and Heatherton, 1996). Proposition 3 describes the behavioral consequences of regulatory loss: reduced evaluative pressure is associated with less selective and less effortful contributions, including increased low-quality voice and reduced investment in performance-critical tasks (Karau and Williams, 1993; Lerner and Tetlock, 1999; Frink and Klimoski, 1998). The full dual-pathway model, including both pathways, their behavioral consequences, and the boundary conditions that moderate their relative dominance, is presented in Figure 1.

## The regulatory loss pathway

The regulatory loss pathway operates through three behavioral channels, each linking the attenuation of evaluative pressure to consequences for individual and group performance.

### Filtering loss

When individuals anticipate social evaluation, they engage in ongoing self-monitoring: screening contributions for relevance, calibrating timing, and adjusting expression to the perceived expectations of the audience (Goffman, 1959; Schlenker and Leary, 1982). The psychological safety literature typically frames this process as suppression, a mechanism through which useful information is withheld out of fear. That characterization is accurate, but incomplete. The same filtering process that screens out valuable contributions also screens out those that are irrelevant, premature, or poorly considered. When psychological safety substantially reduces the perceived cost of speaking, both functions are weakened simultaneously. Consider two officers in the same planning briefing. One has spotted a potential flaw in the plan’s assumption about enemy positioning. The other wants to raise a risk the plan already addresses. Before speaking, each asks himself the same questions: is this worth the group’s time, and is this the moment to raise it. When the cost of speaking is high, both stay silent, and the flaw survives into execution. When the cost of speaking is near zero, both speak, and the briefing spends its limited time on a point already settled. Self-monitoring cannot suppress only the redundant contribution, because the officer doing the monitoring does not know in advance which one he holds.

High psychological safety therefore produces more voice that is more broadly distributed and less filtered. In environments with the capacity to absorb additional input, this may be largely costless. In environments where teams must process multiple streams of information under time pressure, increased volume without corresponding selectivity can create substantial costs. Information overload is a well-documented source of performance decrement in organizational settings (Eppler and Mengis, 2004), and teams operating near their processing capacity are particularly vulnerable. The signal-to-noise problem that emerges under high psychological safety reflects a structural consequence of removing the selection pressure that evaluative risk normally provides. Military planning environments exemplify well this dynamic. Commanders welcome broad input while a plan is being formed and restrict it once the plan is being finalized, because the same contribution that sharpens deliberation disrupts synthesis. In these contexts, the filtering that high psychological safety removes is part of what makes collective reasoning functional.

### Effort withdrawal

The effects of evaluative pressure on effort arise in anticipation of assessment rather than in response to it. Tetlock (1992) suggested that individuals who expect to justify their judgments invest more cognitive effort in forming them and are more resistant to cognitive shortcuts. Lerner and Tetlock (1999) refined this further, showing that pre-decisional accountability to an audience with unknown views produces the most effortful and nuanced reasoning, while post-decisional accountability tends to produce more defensive responses. Extreme levels of psychological safety, particularly when organizational accountability structures are also weak, may attenuate this anticipatory dynamic. When individuals do not expect their contributions to be scrutinized, the motivation to invest in preparation declines. The consequences are typically subtle: contributions that are less developed, greater reliance on others to refine input, reduced attention to timing and precision. Karau and Williams (1993), in their meta-analytic review of social loafing, identified reduced evaluation potential as one of the most consistent predictors of effort withdrawal in collective tasks, an effect amplified in larger groups and lower task visibility conditions, both of which characterize many organizational team settings. In military units, operational readiness depends on norms of preparation and precision sustained partly through the anticipation of evaluation. Commanders assess, peers observe, and after-action reviews hold individuals accountable for the quality of their contributions. When psychological safety lowers the perceived stakes of individual contribution and no accountability mechanism compensates, that motivation may weaken. The observable result is less preparation before meetings, opinions offered without having read the material, and half-formed suggestions passed to the group to finish, because the expected cost of a weak contribution has fallen.

### Norm erosion

The third channel is norm erosion, and it is arguably the most consequential in high-stakes settings. Team norms encode solutions to coordination problems, specifying who raises what, through which channel, and at what point in the task (Feldman, 1984; Bettenhausen and Murnighan, 1991; Weick and Sutcliffe, 2015). These norms are maintained partly by the interpersonal cost of violating them. Psychological safety lowers that cost, and adherence can weaken with it. Pearsall and Ellis (2011) provide direct evidence of such weakening. In teams with utilitarian ethical orientations, higher psychological safety predicted more unethical behavior because the perceived cost of norm violation had fallen. When the norms in question are dysfunctional, weakened adherence is a gain. When they carry coordination solutions, it is a loss, because mistimed or misdirected communication has operational costs. A surgical example illustrates the point. Mid-procedure, a junior resident notices a discrepancy in the instrument count, a legitimate concern that the protocol assigns to the circulating nurse and the next scheduled count. In a team where psychological safety is high but the norm has weakened, the resident instead raises it across the sterile field during a difficult dissection. The willingness to speak is what the enabling pathway predicts, yet the interruption added risk without adding anything the protocol would not have surfaced. The same dynamic could be observed outside high-stakes settings. In a product team with high psychological safety but no clear norm distinguishing planning from execution, a team member flagging an optimistic sprint estimate during planning is exactly the contribution the enabling pathway predicts. The same openness can also let stand-ups become a venue for repeatedly reopening settled estimates, or for updates offered without preparation, where the team has no clear expectation that contributions should be prepared and that finalized decisions should not be casually reopened. The first instance is a legitimate concern raised at the right moment, while the second carries the same willingness to speak with none of its value.

### Collective consequences

At the collective level, the three channels are likely to converge. Unfiltered contributions add informational noise, effort withdrawal leaves quality control to fewer team members, and norm erosion removes the structure that keeps action coordinated. Together they produce process loss, the gap between what a group could achieve and what it actually achieves given how its members interact (Steiner, 1972; Hackman, 2002). How severe these effects become depends on task demands, the strength of existing accountability structures, and how much performance relies on disciplined rather than simply expressive communication. In environments where coordination is loose and stakes are moderate, regulatory loss may be largely invisible. In environments where timing and precision are operationally necessary, its consequences are likely to be significant.

## When psychological safety helps and when it hurts

If psychological safety both enables expression and weakens the regulation of it, its net effect on performance depends on context. The relevant conditions, features of the task, the team, and the organizational environment, have not been systematically specified in the existing literature. The question is no longer whether psychological safety is beneficial, but when, for whom, and under what conditions. The following sections specify these conditions and formalize them as testable propositions.

### Voice quantity vs. quality

One of the most consequential simplifications in the psychological safety literature is the treatment of voice as a unitary construct. The main assumption is that increased voice is beneficial, and that psychological safety’s well-documented effects on voice frequency are therefore straightforwardly positive. Voice frequency and voice quality are analytically distinct dimensions that do not necessarily move together, and conflating them has obscured an important dynamic. Van Dyne and LePine (1998) established voice as a distinct extra-role behavior defined by its constructive and change-oriented character, suggesting from the outset that not all speaking up is equivalent. Liang et al. (2012) distinguished promotive from prohibitive voice and demonstrated that the two forms have different antecedents and consequences, and Morrison's (2011) integrative review reached a similar conclusion: treating voice as a single positive behavior hides substantial variation in its effects. More directly relevant to the present argument, research on group brainstorming has consistently found that removing evaluation apprehension increases the quantity of ideas generated without improving their average quality (Diehl and Stroebe, 1987; Paulus and Yang, 2000). Thus, reducing evaluative pressure may increase the volume of contributions while simultaneously reducing the selectivity with which they are offered.

Recent empirical work reinforces the distinction between voice quantity and quality. Brykman and Raver (2021) found that peers and managers evaluate employees on the quality of what they say over and above how often they say it. In a subsequent field study of an organizational idea platform, voice message quality predicted managerial enactment of ideas, whereas voice frequency was negatively related to peer endorsement (Brykman and Raver, 2023). Meta-analytic evidence further shows that voice and silence are empirically independent constructs and that psychological safety relates more strongly to reduced silence than to increased voice (Sherf et al., 2021), which suggests that raising psychological safety cannot be assumed to raise the constructive voice the literature has in mind.

This suggests that psychological safety has a stronger positive relationship with voice quantity than with voice quality, resulting in increased variability in the usefulness of contributions as psychological safety rises. Teams operating under high psychological safety are likely to generate more input, but that input will reflect a wider distribution of quality, with both more genuinely valuable contributions and more redundant or poorly calibrated ones. The net effect depends on the team’s capacity to discriminate between them. Edmondson and Bransby (2023) explicitly flag voice quality as an underexplored dimension that future research needs to address, lending support to the theoretical gap this proposition identifies.

### Task phase

A second source of divergence is the temporal structure of complex work. Most organizational tasks do not place uniform demands on communication throughout their duration; they unfold across phases that differ in what kind of input is valuable and how much coordination matters relative to exploration. Research on group development (Tuckman, 1965; Gersick, 1988) and team cognition (Kozlowski and Ilgen, 2006) has long recognized this structure, but its implications for psychological safety remain undeveloped.

The distinction between deliberation and execution phases matters most. During deliberation, the task is to generate and evaluate information, and the enabling effects of psychological safety are at their most valuable. Voice is useful, challenge is productive, and a contribution withheld out of fear is a genuine loss. This is the context in which Edmondson's (1999) original research was conducted and in which the enabling pathway operates most cleanly. During execution, coordinated action under time pressure requires clarity, speed, and adherence to protocol. The value of additional input now depends on its precision and timing, and communication discipline predicts effective execution better than communication volume (Salas et al., 2015; Entin and Serfaty, 1999). Military planning reflects the same division: input is deliberately maximized while courses of action are being developed and deliberately restricted once execution begins (Department of the Army, 2019a).

When psychological safety remains uniformly high across phases, communication behavior can fall out of alignment with task requirements. The openness that served planning introduces noise and ambiguity during execution, where coordination failures are most costly. The costs of unstructured communication in this phase are documented in Snook’s (2000) analysis of the friendly fire shootdown of US Black Hawk helicopters over northern Iraq, in which ambiguous communication norms and insufficient command clarity contributed to a catastrophic misidentification. Psychological safety without phase-sensitive communication norms is an incomplete solution to the problem of organizational silence.

### Non-linear effects

The interaction of enabling and regulatory mechanisms also points toward the possibility of nonlinear relationships between psychological safety and performance, a possibility the field has been slow to theorize despite early empirical signals. Eldor et al. (2023) provided the most direct evidence to date, finding across five studies that the relationship between psychological safety climate and in-role performance follows an inverted-U pattern: moderate levels predict the best outcomes, while very high levels are associated with performance decline, particularly for routine tasks. Their framework suggests that very high safety climates undermine the attention and discipline that routine tasks require, and they show that collective accountability buffers the decline. What remains unspecified is the process through which safety erodes discipline in the first place, and why the erosion becomes visible only at high levels.

The dual-pathway framework offers a more precise explanation. The enabling benefits of psychological safety are likely to exhibit diminishing returns once the primary barriers to participation have been removed. The first substantial reduction in interpersonal risk produces the largest gains, bringing previously suppressed information into deliberation and encouraging the error-reporting that Edmondson (1999) originally documented. Further reductions add less, because the important barriers are already gone and what remains to be enabled is of lower marginal value. The regulatory costs follow a different trajectory. Filtering, accountability-driven effort, and norm adherence erode gradually as evaluative pressure declines, and their erosion becomes visible in performance only at high levels of psychological safety. The back-loading of these costs is consistent with how evaluation potential operates: self-monitoring responds to the presence of an evaluative prospect more than to its intensity, which is why effort losses emerge when contributions become unidentifiable rather than scaling smoothly with identifiability (Karau and Williams, 1993). The asymmetry between these two curves produces the inverted-U that Eldor et al. (2023) observed and explains why performance declines at high rather than moderate levels of safety. It also accounts for their moderation result: collective accountability buffers the decline because it reinstates the anticipatory evaluative pressure that high safety attenuates.

The argument parallels work on optimal distinctiveness (Brewer, 1991) and on exploration and exploitation in organizational learning (March, 1991), both of which describe resources that are beneficial at moderate levels and counterproductive in excess. In the present case, the mechanism is specific. At moderate levels of psychological safety, regulatory constraints remain strong enough to discipline expression without suppressing it; at very high levels, they have eroded to the point where the system operates with insufficient regulatory structure. The strength of external regulatory mechanisms should moderate this nonlinearity. Where accountability structures, performance monitoring, and coordination norms are strong and independent of psychological safety, the regulatory loss is partially compensated; where they are weak, the costs of very high safety are more severe.

These arguments generate three propositions. Proposition 4 captures the voice asymmetry: psychological safety has a stronger positive relationship with voice quantity than with voice quality, such that increases in voice under high psychological safety include both more genuinely valuable contributions and more redundant or poorly calibrated ones. Proposition 5 addresses task-phase contingency: the positive effects of psychological safety on team effectiveness are stronger during deliberation-oriented phases, while the negative effects of very high psychological safety are more likely to emerge during execution-oriented phases where coordinated action and communication discipline are required. Proposition 6 formalizes the nonlinear relationship: the association between psychological safety and team performance follows an inverted-U pattern, with moderate levels associated with optimal outcomes and very high levels associated with performance decline, particularly in contexts characterized by high coordination demands, routine task structures, or weak external accountability.

## Boundary conditions

The balance between enabling and regulatory effects is shaped by the broader regulatory structures within which psychological safety operates. Where alternative sources of evaluative structure are strong, the regulatory costs of reduced interpersonal risk may be absorbed or compensated. Where they are absent, those costs are more likely to translate into observable performance decline. This section develops three boundary conditions that shape this dynamic: formal accountability structures, leader structuring, and the internalization of norms through professional identity and shared experience.

### Formal accountability

The most direct counterweight to reduced evaluative pressure is institutionalized accountability. Within the model, this condition operates primarily on the effort withdrawal channel, because anticipatory accountability is the mechanism through which evaluative pressure sustains preparation and deliberative investment. Anticipated accountability promotes more effortful and defensible reasoning, particularly when it is pre-decisional and the audience’s views are unknown (Tetlock, 1992; Lerner and Tetlock, 1999). Reviewing the organizational literature, Frink and Klimoski (1998) found that formal accountability systems shape performance and decision quality through anticipatory mechanisms that operate largely independently of interpersonal climate. Formal accountability can therefore partially substitute for the evaluative pressure that psychological safety attenuates. When individuals know that their contributions will be assessed, recorded, or reviewed, some of the regulatory functions of interpersonal risk are reproduced through structural means even in psychologically safe environments. Nuclear power plants operate with layered audit and reporting systems that sustain evaluative pressure regardless of the interpersonal climate within work groups (Schulman, 2020; Reason, 1997). Commercial aviation maintains incident reporting systems and simulator-based evaluation that create a continuous accountability infrastructure around crew performance. The Aviation Safety Reporting System, administered by NASA, illustrates the design principle directly: reporters receive confidentiality and limited protection from enforcement action, while the evaluability of incident data is preserved at the system level (Leape, 2002). Military organizations build these structures most extensively. Formal evaluation reports, command inspections, after-action reviews, and readiness assessments create accountability that operates continuously and largely independently of team dynamics (Hannah et al., 2009; Wong et al., 2003). A junior officer in a high-psychological-safety environment still knows that the quality of their contributions will be visible, recorded, and consequential. The interpersonal cost of speaking has been reduced, but the professional cost of contributing poorly has not.

### Leader structuring

The second boundary condition concerns how leaders structure the conditions under which psychological safety is exercised. Within the model, this condition operates primarily on the filtering channel, because phase-sensitive structuring restores the selectivity and timing that reduced evaluative pressure weakens. An environment in which all voice is equally welcome at all moments is a different system from one in which leaders signal when challenge is expected, how dissent should be expressed, and when execution requires adherence to protocol. Weick and Sutcliffe (2015) describe effective leadership in high-reliability organizations as genuine openness to bad news combined with clear structuring of when and how that news should surface. Salas et al. (2015) found that the most effective leaders in high-stakes environments combine participative and directive styles, using the former during planning and the latter during execution, the task-phase contingency developed above.

Institutional practice reflects the same design. The sterile cockpit rule introduced earlier is a formal specification of when open exchange gives way to communication discipline. The surgical time-out embedded in the WHO Surgical Safety Checklist creates a defined moment at which any team member is expected to raise concerns, and its implementation reduced complications and mortality across eight hospitals in eight countries (Haynes et al., 2009). Mission command in military doctrine creates space for voice and initiative within clearly defined commander’s intent, bounding that space with constraints that preserve unity of effort (Department of the Army, 2019b; Wong et al., 2003). Lorinkova et al. (2013) found a parallel dynamic in civilian teams: empowering leadership outperformed directive leadership over time, but only as teams developed the shared understanding needed to exercise voice productively. Across these contexts, the value of psychological safety depends on whether leaders have built the structures that give expression its coordination value.

### Internalized norms

The third boundary condition is the most theoretically distinctive. It describes a situation in which the regulatory loss pathway is attenuated without formal accountability or active leader structuring, because regulatory discipline has been internalized through shared experience and professional identity. In the model, this condition operates primarily on the norm erosion channel, because internalized standards do not depend on the interpersonal costs that psychological safety removes.

Groups with highly developed shared mental models coordinate implicitly, anticipating each other’s actions and adjusting behavior without explicit communication (Kozlowski and Ilgen, 2006; Cannon-Bowers and Salas, 2001). Coordination in such teams does not depend on external regulatory pressure because individuals have internalized standards of appropriate behavior that are self-sustaining. Entin and Serfaty (1999) documented this directly: experienced military teams under stress reduced communication volume while maintaining performance, because shared mental models allowed them to infer what others needed without being told. External evaluative pressure had become largely redundant.

Professional military units, surgical teams, and experienced emergency response organizations often combine interpersonal openness with behavioral discipline. Work on leadership in extreme contexts suggests that such units sustain discipline through standards internalized under conditions where errors carry severe consequences (Hannah et al., 2009). Psychological safety removes the interpersonal cost of speaking. The internalized professional cost of contributing poorly or at the wrong moment remains. Teams with weak norms, limited shared experience, or low professional cohesion are considerably more vulnerable. In such teams, external evaluative pressure may be the primary mechanism sustaining communicative discipline, and its attenuation through psychological safety is more likely to produce the process losses described earlier. This is particularly relevant for newly formed teams, teams operating across professional or cultural boundaries, and organizations where professional socialization is weak, conditions that characterize a significant proportion of the military and emergency response contexts where high psychological safety is currently being advocated.

These boundary conditions share a principle. Formal accountability, leader structuring, and internalized norms each provide regulatory structure that can compensate for the evaluative pressure psychological safety attenuates. Each condition attenuates most directly the channel it targets, and the regulatory loss pathway dominates when all three are weak. This yields the final proposition: the negative effects of psychological safety on team effectiveness are attenuated when regulatory structures are present and amplified when they are absent. Together with the preceding propositions, this constitutes the dual-pathway model, summarized in Table 1.

**Table 1:** Summary of theoretical propositions.

Proposition	Statement	Key references
P1	Psychological safety is positively associated with willingness to engage in voice and interpersonal expression.	Edmondson (1999), Frazier et al. (2017)
P2	Psychological safety is negatively associated with interpersonally based evaluative pressure, resulting in reduced self-regulation of communication and behavior.	Tetlock (1992), Carver and Scheier (1998), Baumeister and Heatherton (1996)
P3	Reduced evaluative pressure is associated with less selective and less effortful contributions, including increased low-quality voice and reduced investment in performance-critical tasks.	Karau and Williams (1993), Lerner and Tetlock (1999)
P4	Psychological safety has a stronger positive relationship with voice quantity than with voice quality, such that increases in voice under high psychological safety include both more valuable and more redundant contributions.	Diehl and Stroebe (1987), Paulus and Yang (2000), Brykman and Raver (2021), Edmondson and Bransby (2023)
P5	The positive effects of psychological safety are stronger during deliberation-oriented task phases, while negative effects are more likely during execution-oriented phases requiring coordinated action.	Salas et al. (2015), Entin and Serfaty (1999), Gersick (1988)
P6	The relationship between psychological safety and performance follows an inverted-U pattern, with very high levels associated with performance decline under high coordination demands or weak accountability.	Eldor et al. (2023)
P7	The negative effects of psychological safety are attenuated when regulatory structures are present and amplified when they are weak or absent.	Tetlock (1992), Frink and Klimoski (1998), Hackman (2002), Eldor et al. (2023)

P1 and P2 establish the dual-pathway mechanism. P3 and P4 specify behavioral consequences of regulatory loss. P5 identifies task phase as a contingency. P6 formalizes the nonlinear performance relationship. P7 specifies boundary conditions.

## Hierarchy and power

The framework developed so far treats regulatory structure as something organizations build through accountability systems, leadership practices, and internalized norms. Hierarchy warrants separate treatment because it is the most pervasive regulatory structure in organizational life and because it shapes both pathways simultaneously. Hierarchies are self-reinforcing orders that establish who may speak, in what sequence, and with what authority (Magee and Galinsky, 2008). In the settings this paper is concerned with, hierarchy is also formal and often legally codified, which makes its interaction with psychological safety a necessary part of the analysis. Hierarchy is not a fourth boundary condition; it is the context within which the three boundary conditions operate, shaping both pathways at once. Hierarchy shapes the enabling pathway by distributing interpersonal risk unevenly. The risk that psychological safety reduces is not experienced uniformly across ranks. Individuals with a low sense of power are substantially more likely to remain silent, an effect that operates through inhibition processes closely related to the evaluative pressure described in the regulatory loss pathway (Morrison et al., 2015). High-power members face weaker inhibition and therefore gain comparatively little from further increases in psychological safety, while low-power members may require considerable safety before speaking at all. Interventions that raise psychological safety in strongly hierarchical settings therefore change the distribution of voice across ranks as well as its overall volume.

This is consistent with evidence that leader inclusiveness matters most for lower-status professionals (Nembhard and Edmondson, 2006). Hierarchy shapes the regulatory pathway through a different mechanism. Clear rank structures specify when input is expected, through which channel it should travel, and who owns the decision. This clarity performs regulatory work of the kind the framework attributes to evaluative pressure: it filters contributions, times them, and bounds them. An order that defines the limits of a task also defines the limits of appropriate contribution, and members of order-driven cultures often experience this clarity as a source of security rather than suppression, because it removes ambiguity about what is expected and what is at stake. In this respect, formal hierarchy can substitute for part of the interpersonal evaluative pressure that psychological safety attenuates, which helps explain why rank-based organizations can sustain high psychological safety and disciplined communication at the same time.

Hierarchy is not uniformly regulatory, however. Meta-analytic evidence indicates that hierarchy has a negative average effect on team effectiveness and that this effect operates through conflict-enabling states, with the damage concentrated where hierarchical arrangements are unstable or contested (Greer et al., 2018). This evidence suggests a distinction between two hierarchical conditions. Legitimate and stable hierarchy functions as regulatory structure, channeling voice without suppressing it. Contested hierarchy politicizes communication: members use voice to claim status and silence to avoid exposure, and under these conditions neither pathway operates as the model describes, because contributions no longer carry information alone. These considerations qualify the boundary conditions developed above. Formal accountability, leader structuring, and internalized norms all operate within a hierarchical context that determines who can draw on them and at what interpersonal cost. They also mark a limit of the present framework. The model specifies how evaluative pressure regulates the quality and timing of contributions, but it does not yet specify how power asymmetries alter the experience of that pressure across ranks. A full integration of the power literature with the dual-pathway model is a distinct theoretical undertaking.

## Discussion

### Theoretical contributions

The dual-pathway framework developed in this paper addresses an imbalance in how psychological safety has been theorized. The enabling effects of the construct are well documented (Frazier et al., 2017; Newman et al., 2017; Edmondson and Bransby, 2023). What has received comparatively little attention is the set of regulatory structures the same mechanism may weaken. The scope of this argument should be stated precisely. Nothing in the framework disputes the value of psychological safety. The evidence that interpersonal fear suppresses error reporting, learning, and voice is extensive (Edmondson, 1999; Frazier et al., 2017), and in the settings this paper examines, the costs of silence have been severe. The claim is conditional: at very high levels, and where compensating regulatory structures are weak, the same mechanism that removes the fear of speaking also removes part of what disciplines it. A framework that specifies these conditions is an argument for deploying psychological safety well, and against deploying it blindly.

The first contribution is conceptual. Psychological safety has largely been theorized as an unambiguously positive climate construct, with the dominant research agenda organized around identifying its antecedents and documenting its beneficial consequences (Kahn, 1990; Baer and Frese, 2003; Nembhard and Edmondson, 2006). This paper reframes it more neutrally as a mechanism of interpersonal risk reduction whose effects depend on what that reduction does to the broader regulatory environment in which teams operate. This shift from outcome-based to mechanism-based theorizing clarifies why the same construct produces enabling effects in some contexts and dysregulating effects in others, and it provides a theoretical foundation for the contingent relationships the existing literature has documented empirically but not fully explained (Edmondson and Lei, 2014; Eldor et al., 2023). It responds directly to Edmondson and Bransby’s (2023) observation that psychological safety research has matured to a point where boundary conditions and unintended consequences deserve more systematic theoretical attention.

The second and most substantive contribution is the specification of the regulatory loss pathway. Drawing on self-regulation theory, accountability research, and the social loafing literature, the paper shows that evaluative pressure both suppresses and structures behavior. Individuals who anticipate social evaluation filter their contributions more carefully (Schlenker and Leary, 1982), invest more cognitive effort in preparing them (Tetlock, 1992; Lerner and Tetlock, 1999), and calibrate their timing more precisely to task demands (Goffman, 1959). When psychological safety attenuates this anticipatory pressure, these regulatory processes are weakened alongside the fear that the literature rightly identifies as a barrier to voice. The result is a shift both in how much individuals contribute and in how disciplined those contributions are. This mechanism provides an explanation for the nonlinear relationships between psychological safety and performance that have recently begun to emerge empirically (Eldor et al., 2023), moving the explanation beyond the observation that too much safety reduces motivation toward a more precise account of which cognitive and behavioral processes are responsible.

The third contribution is to situate psychological safety within the organization’s wider regulatory structures. Rather than treating the construct in isolation, the framework positions it alongside formal accountability, leadership practices, and the internalized norms produced by professional identity and shared experience. This addresses an unresolved empirical pattern: high psychological safety coexists with disciplined, high-performing teams in some organizations and with coordination failures in others. The level of psychological safety is not the operative variable. What matters is whether the surrounding regulatory structures remain strong enough to discipline the expression it enables. Mission command, discussed above, is the institutional case in point: openness at the interpersonal level, evaluative pressure preserved at the systemic level, with effectiveness contingent on the surrounding accountability and norm structures (Department of the Army, 2019b; Wong et al., 2003; Hannah et al., 2009). The framework goes beyond existing boundary condition accounts, including Edmondson’s (2018) two-by-two model, by specifying the mechanism: psychological safety attenuates the interpersonal sources of anticipatory evaluative pressure, which is why structural sources such as formal accountability can compensate for the loss. The fourth contribution is the development of context-sensitive propositions that specify when psychological safety helps and when it hurts. By distinguishing between voice quantity and voice quality, between deliberation and execution task phases, and between environments with strong versus weak regulatory structures, the framework offers a more precise account of psychological safety’s contingent effects than the existing literature provides, responding directly to calls for greater attention to boundary conditions and unintended consequences (Edmondson and Bransby, 2023; Eldor et al., 2023).

Another contribution is the integration of three theoretical traditions that have developed largely in parallel: self-regulation theory (Carver and Scheier, 1998; Baumeister and Heatherton, 1996), accountability research (Tetlock, 1992; Frink and Klimoski, 1998), and the social loafing literature (Latané et al., 1979; Karau and Williams, 1993). Each tradition has examined how evaluative pressure shapes behavior, and individual connections to psychological safety have surfaced in prior work (e.g., Pearsall and Ellis, 2011; Eldor et al., 2023). What has been missing is a systematic integration: the dual-pathway framework specifies how the three mechanisms relate, connects each to a distinct behavioral channel, and shows how they compound at the team level.

### Relationship to existing literature

The dual-pathway model extends emerging empirical work on the limits of psychological safety by providing the mechanistic account those studies have lacked. Eldor et al. (2023) demonstrated that the relationship between psychological safety climate and in-role performance follows an inverted-U pattern, and that collective accountability buffers the decline at high levels. The dual-pathway model explains both results. The nonlinearity emerges because enabling benefits exhibit diminishing returns once primary barriers to participation are removed, while regulatory costs accumulate more gradually and become visible in performance outcomes only at higher levels of safety. This is a specific prediction about the asymmetric scaling of two competing mechanisms, distinguishing the present framework from generic excess arguments (Pierce and Aguinis, 2013) and making it directly testable.

The framework also brings the psychological safety literature into productive contact with the accountability and self-regulation traditions, which have largely developed in parallel. Evaluative pressure does more than deter potential speakers; it shapes how carefully they prepare, filter, and time their contributions before speaking at all. Tetlock (1992), Carver and Scheier (1998), and Baumeister and Heatherton (1996) each provide theoretical tools that showcase this mechanism, and Frink and Klimoski’s (1998) review found that formal evaluative structures shape performance and decision quality through anticipatory mechanisms that operate largely independently of interpersonal climate, directly supporting the compensatory moderation argument developed in Proposition 7. Hall et al.’s (2017) review similarly finds that felt accountability operates as a regulatory system distinct from interpersonal climate, which is what allows it to compensate when climate-based evaluative pressure weakens.

The paper contributes to high-reliability organization research by clarifying when interpersonal openness is compatible with operational discipline. Roberts (1990) identified structural redundancy and formal accountability as core features of reliable systems, features the present framework identifies as compensatory regulatory mechanisms. La Porte and Consolini (1991) found that reliability under pressure depended on a combination of strong professional norms, formal authority structures, and genuine openness to safety-relevant information, the combination of regulatory structures the present framework identifies as optimal.

The framework also complicates the organizational silence literature’s prescription that silence is best addressed through greater psychological safety (Edmondson, 1999; Morrison and Milliken, 2000). The regulatory functions of anticipated evaluation are embedded in the same social dynamics that produce silence, and dismantling those dynamics entirely may solve one problem while creating another. What the framework suggests is that interventions designed to address silence need to be more carefully calibrated than a blanket increase in psychological safety, consistent with Detert and Edmondson’s (2011) finding that voice behavior is shaped by a complex mix of interpersonal, contextual, and strategic considerations that no single climate intervention can fully address. Morrison’s (2023) review of the past decade of voice research reaches a compatible conclusion: the field has moved from asking whether employees speak up toward asking when, how, and with what consequences, and the dual-pathway model provides a mechanism-level account of that shift.

Finally, the framework joins a body of work showing that ostensibly positive constructs can produce negative outcomes under specifiable conditions (Fineman, 2006; Hackman, 2009; Bolino et al., 2013). Research on proactive work behavior has made the same move. Bolino et al. (2010) argued that expecting employees to behave proactively carries costs for the employees themselves, and Strauss et al. (2017) showed empirically that proactive behavior increases job strain when it is enacted under pressure and obligation rather than autonomous motivation, a self-regulatory cost structure closely related to the one theorized here. The wise proactivity framework draws the same conclusion at the level of enactment: the value of a self-initiated behavior depends on how, when, and toward what it is directed (Wang et al., 2025). Bolino et al.’s (2013) analysis of the dark side of organizational citizenship behavior follows the same logic for citizenship. The present framework attempts this for psychological safety. Its value depends on the surrounding regulatory structures, and specifying that dependence is more useful than restating the construct’s general benefits (Grant and Schwartz, 2011; Pierce and Aguinis, 2013).

### Practical implications

Organizations should not aim to maximize psychological safety in isolation. The goal is to balance interpersonal openness with sufficient regulatory structure, and the two are more complementary than the dominant discourse has suggested. Framing evaluative pressure purely as a threat has led organizations to pursue psychological safety in ways that leave its regulatory costs unaddressed (Edmondson and Bransby, 2023).

The most concrete implication concerns organizational design. The aviation, nuclear, and military examples examined throughout this paper share a structural feature: individuals feel genuinely safe to speak while remaining accountable for the quality of their contributions. Mandatory incident reporting systems protect reporters from retaliation while preserving the evaluability of their contributions (Leape, 2002). After-action reviews temporarily set aside rank while holding contributions to standards of substance; the United States Army’s version is doctrinally institutionalized, conducted after significant training and operational events, and structured around fixed questions that direct attention to performance. The sterile cockpit rule and the surgical time-out described earlier offer equally concrete templates, one restricting communication during execution and the other mandating a structured opening for it before execution begins. Performance evaluation systems that assess reasoning quality and decision processes, and not only outcomes, create the anticipatory accountability that sustains disciplined contribution in psychologically safe environments (Tetlock and Gardner, 2015). Organizations seeking the benefits of psychological safety without its regulatory costs should study these designs; the mechanisms involved are structural, and cultural interventions alone do not reproduce them.

For leaders, the most important skill is calibration. This means signaling when challenge is expected and how it should be expressed (Detert and Burris, 2007), distinguishing phases of work where voice is an asset from phases where coordination takes priority (Salas et al., 2015; Entin and Serfaty, 1999), and modeling the combination of openness and accountability the framework identifies as optimal. Mission command offers a well-developed practical template that organizations outside the military could adapt (Wong et al., 2003). For teams, the internalization of professional norms and shared standards is part of the structural foundation of performance. Teams that rely exclusively on external accountability to sustain coordination are more vulnerable to regulatory loss than teams in which professional identity and shared experience have produced internalized standards. Investments in team development, professional socialization, and shared mental models are necessary complements to psychological safety (Cannon-Bowers and Salas, 2001; Kozlowski and Ilgen, 2006).

### Future research

The most fundamental gap in the existing literature is the treatment of voice as a unitary construct. Most empirical work on psychological safety uses measures that capture willingness to speak without distinguishing the quality, timing, or relevance of what is said, which makes it impossible to test whether psychological safety increases functional voice, dysfunctional voice, or both. Measures separating voice quality from frequency now exist (Brykman and Raver, 2021) but have not been connected to psychological safety. Future research should examine whether quantity and quality respond differently to increases in psychological safety, particularly where communication discipline matters for performance.

A second gap concerns boundary conditions. Most studies treat psychological safety as a main effect and have not systematically examined the moderating role of accountability structures, leader structuring, or norm strength. The compensatory moderation argument in Proposition 7 generates specific predictions about when high psychological safety produces positive versus negative outcomes, and these are directly testable in field settings where accountability infrastructure varies naturally across teams. Experimental designs that manipulate psychological safety and accountability independently would allow the most direct tests of the dual-pathway mechanism.

Third, the contexts in which psychological safety has been studied remain narrow. The construct has been examined primarily in healthcare, education, and corporate settings with moderate stakes and relatively flat hierarchies. Military units, emergency response organizations, and other high-reliability contexts remain underrepresented despite being theoretically important boundary cases. Research in these settings would test the generalizability of established findings and would provide the most direct evidence for the regulatory loss pathway, since coordination demands and accountability structures are unusually visible and measurable there. A fourth direction concerns hierarchy and power. The analysis above treats stable, legitimate hierarchy as a regulatory structure and contested hierarchy as a source of political dynamics that alter what voice signals. This distinction is testable: the performance effects of psychological safety should vary with the legitimacy and stability of the surrounding rank structure, and voice should respond to psychological safety more strongly at lower levels of power (Morrison et al., 2015; Greer et al., 2018). Designs that measure voice separately by rank, rather than aggregating it to the team level, would allow these predictions to be examined directly. Finally, the field needs better measurement of the environment itself. Existing psychological safety scales capture perceived interpersonal safety but do not separate it from perceived evaluative pressure, treating as one dimension what the dual-pathway model requires to vary independently. Without instruments that make this distinction, the model cannot be tested with precision. Developing and validating such measures would be a methodological contribution in its own right.

## Conclusion

Psychological safety has earned its place at the center of organizational research by making visible the costs of silence and the value of interpersonal openness (Edmondson, 1999; Kahn, 1990; Morrison and Milliken, 2000). The argument advanced here extends that contribution. Reducing interpersonal risk enables expression, and at sufficiently high levels of safety it also weakens the regulatory processes on which coordinated and timely decisions depend (Feldman, 1984; Hackman, 2002; Weick and Sutcliffe, 2015). The practical stakes are visible in settings such as operational planning or surgery, where whether people speak up matters, and so do the content and timing of what they say. Evidence from military, aviation, and high-reliability settings indicates that the most effective systems calibrate psychological safety, cultivating interpersonal openness while preserving evaluative discipline at the systemic level. Organizations that treat psychological safety as a quantity to be maximized risk weakening the regulatory processes that give voice its value.
